# Quality of Life and Autonomy in Patients with Intermittent Bladder Catheterization Trained by Specialized Nurses

**DOI:** 10.3390/jcm10173909

**Published:** 2021-08-30

**Authors:** Blanca Fernandez-Lasquetty Blanc, Julián Rodríguez-Almagro, Carlos Lorenzo-García, Elena Alcaraz-Zomeño, Guadalupe Fernandez-Llorente, Montserrat Baixauli-Puig, María Victoria Martín-Bermejo, Francisco Estudillo-González, Maria Angustias Ortega-Checa, Vicenta Lluesma-Martinez, Guillermina Ferrández-Franco, Begoña Benito-Santos, Mónica Rodríguez-Díaz, Arancha Torres-Bacete, María Carmen Guerrero-Andrades, Mario Pierre Louis-Lauture, Isabel Jiménez-Mayorga, Rosario Serrano-Abielar, María Asunción Garrido-Mora, Francisco Barcia-Barrera, Gemma Asensio-Malo, Montserrat Morcillo-Marín, Silvia Tendero-Ruiz, Antonio Hernández-Martínez

**Affiliations:** 1 Enferconsultty, 28006 Madrid, Spain; blancafl@enferconsultty.es; 2 Department of Nursing, Physiotherapy and Occupational Therapy, Ciudad Real Faculty of Nursing, University of Castilla-La Mancha, 13071 Ciudad Real, Spain; antomatron@gmail.com; 3 Department of Nursing, Hospital Universitario Clínico San Carlos, 28040 Madrid, Spain; carloslorenzogarcia@yahoo.es; 4 Department of Nursing, Hospital Universitario Ramón y Cajal, 28034 Madrid, Spain; elenaalcaraz85@hotmail.com; 5 Department of Nursing, Hospital Universitario Infanta Sofía, 28702 Madrid, Spain; gferllo1967@gmail.com; 6 Department of Nursing, Hospital Universitari Clinic Barcelona, 08036 Barcelona, Spain; MBAIXAUL@clinic.cat; 7 Department of Nursing, Hospital Nacional de Paraplejicos, 45004 Toledo, Spain; ebencas@movistar.es; 8 Department of Nursing, Hospital Universitario Puerto Real, 11510 Cadiz, Spain; pacoestudillo@gmail.com; 9 Department of Nursing, Hospital Universitari I Politecnic La Fe, 46026 Valencia, Spain; maorche@outlook.es (M.A.O.-C.); vicenlluesma@gmail.com (V.L.-M.); 10 Department of Nursing, Hospital General Universitario de Alicante, 03010 Alicante, Spain; guillerminaferrandez@gmail.com (G.F.-F.); bebsan2m@hotmail.com (B.B.-S.); 11 Department of Nursing, Hospital Universitario Virgen de las Nieves, 18014 Granada, Spain; rodriguezmonica09m2@gmail.com; 12 Department of Nursing, Hospital Universitario Infanta Leonor, 28031 Madrid, Spain; atbacete@gmail.com; 13 Department of Nursing, Hospital Universitario Puerta del Mar, 11009 Cadiz, Spain; mcguean@ono.com; 14 Department of Nursing, Hospital Universitario Virgen Macarena, 41009 Sevilla, Spain; mario-pierre-louis@hotmail.es; 15 Department of Nursing, Hospital Universitario Regional de Malaga, 29010 Malaga, Spain; iejmayorga@gmail.com; 16 Department of Nursing, Hospital Universitario Puerta de Hierro Majadahonda, 28222 Madrid, Spain; rosarioserrano.ab@gmail.com; 17 Department of Nursing, Hospital General Universitario de Elche, 03203 Valencia, Spain; asungmora@hotmail.com; 18 Department of Nursing, Hospital Universitario Virgen del Rocio, 41013 Sevilla, Spain; Frbarcia@gmail.com; 19 Department of Nursing, Hospital Universitari de Bellvitge, 08907 Barcelona, Spain; gasensio@bellvitgehospital.cat; 20 Department of Nursing, Institut Guttmann, 08916 Barcelona, Spain; monmoma@hotmail.com; 21 Department of Nursing, Hospital Universitario de Fuenlabrada, 28942 Madrid, Spain; t_silvia_r@hotmail.com

**Keywords:** intermittent bladder catheterization, adherence, self-care, risk factors

## Abstract

Intermittent bladder catheterization (IBC) involves regular urine draining using a catheter, which is removed immediately after urinary elimination. It allows for the patient’s urological health to be managed and their renal function to be preserved, and it promotes autonomy. Compliance with the prescribed number of daily catheterizations, which must be conducted by the patient, and infection prevention measures are crucial. To identify the patients requiring IBC, and to determine their adherence (whether they followed the prescribed guidelines and their difficulty in carrying out the procedure, as well as to assess how the IBC influences their quality of life and state of mind after receiving self-care training from a specialized nurse), we carried out a prospective, multicenter observational study in 24 Spanish hospitals with one month of monitoring and a sample of 99 patients. The sources of information were the patients’ clinical records, the King’s Health Questionnaire, the Mini-Mental State Examination (MMSE), and the hospital anxiety and depression scale (HADS). Descriptive and bivariate statistics were used to analyses the paired data. After recruitment (*n* = 99), 79 patients completed the questionnaire at a mean age of 35.2 years (SD = 20.5 years). In total, 53.5% (53) of the sample consisted of men and 32.3% (32) had neurological damage as the reason for prescription; 67% (67.7) performed self-catheterization and 86.7% adhered to the IBC. After one month of monitoring, a statistically significant improvement in quality of life was observed in all criteria, with the exception of personal relationships (*p* < 0.005), as well as an improvement in anxiety and depression levels (*p* < 0.001). Patients who require IBC show good adherence to the IBC with a significant percentage of self-catheterization. After one month of IBC, a significant improvement in the patients’ quality of life and mood was observed. These results could be attributed to adequate patient training and adequate personalization of the IBC materials by the specialized nurses.

## 1. Introduction

Intermittent bladder catheterization (IBC) is the periodic drainage of urine through a catheter inserted through the urethra into the bladder, or through another surgically accessed channel. Once the catheter is inserted and the urine is drained, it is removed immediately. This treatment allows for the patient’s urological health to be managed and their renal function to be preserved, and it promotes autonomy.

IBC is a critical part of healthcare for individuals with incomplete urinary evacuation that aims to protect the bladder and preserve renal function [1], as well as for individuals with overflow incontinence [2,3,4]. IBC is considered the gold standard in the treatment of neurogenic/neuropathic bladder disorders [5] and has a number of advantages over permanent bladder catheterization, because it reduces adverse events associated with its use, such as urinary tract infections, hematuria, and vesical tenesmus. It also allows the patient to have greater independence and improves factors such as social, work, and school integration, relationships, self-esteem, and quality of life in general [2,4,6].

IBC can be carried out by people of all ages, including elderly people and children from 4 to 5 years of age, under the supervision of an adult. The technique can be performed by other persons or by the patients themselves (self-catheterization), provided they do not have disabilities such as blindness, the absence of perineal sensation, tremors, a mental disability, or paraplegia, which would impede their control over the technique [6,7].

To be able to carry out IBC, the person conducting it needs to be trained by a professional, usually a nurse who is an expert in functional urology and who will not only train them to perform this technique, but will also train them in self-care and in how to include the treatment into their everyday life. Compliance with the prescribed number of daily catheterizations, which must be conducted by the patient, in addition to the infection prevention measures, are crucial.

However, research has shown that there are a number of factors that reduce adherence to IBC, such as the lack of suitable places with sufficient privacy to be able to perform the technique in public establishments, the lack of time, the need to plan ahead for IBC, and the need for help in order to perform the catheterization [4,8,9,10].

It has also been observed that patients who start IBC may not follow the recommendations strictly and may perform less daily catheterizations than recommended, which occurs in other long-term treatments [10], with the complications that this entails. Predicting which patients are most likely to adhere to IBC is, therefore, paramount in preventing future complications.

Variables such as age, sex, urethral sensitivity, pain, general health, quality of life, mobility, and the training provided by nurses experienced in this field to the patient have also been suggested as factors influencing adherence to treatment [10]. However, there is a lack of sufficient studies on the general population who are susceptible to IBC to clearly demonstrate which of these variables plays a decisive role in adherence to IBC treatment [10].

On the other hand, the relationship between IBC and improved quality of life, as well as its emotional impact, has been little studied [11,12], as most studies are qualitative in nature [12,13,14,15,16] and have small sample sizes.

Therefore, the aim of this study was to identify patients who require IBC, determine their adherence and their difficulties in performing the technique, and to assess how IBC affects their quality of life and state of mind in the short term.

## 2. Material and Methods

### 2.1. Design and Selection of Study Subjects

This was a prospective multicenter observational pilot study in 24 hospital facilities in 11 Spanish cities from 15 October 2020 to 15 December 2020. This study received the approval of the Ethics and Clinical Research Committee of CEIC Hospital Clínico San Carlos, with protocol number 19/156-O.

The reference population was made up of patients who perform IBC, treated at facilities participating in the study. The inclusion criteria were that IBC was prescribed for the first time and that the patient was over 18 years old. The exclusion criteria were patients who did not understand the language of the interlocutor or who had cognitive and/or sensory difficulties affecting their understanding of the purpose of the study, as well as their ability to perform IBC, and post-surgical patients.

The study population was made up of patients in the reference population who, having met the inclusion criteria, voluntarily agreed to participate. The withdrawal criteria for the study patients were the patient deciding to withdraw, changes in hospital facility, death during the study, or end of the treatment.

Because several aspects were assessed (adherence to IBC, quality of life, mood), taking into account a prevalence of 50% (the most demanding in sample size), a confidence level of 95%, and an unknown reference population susceptible to requiring IBC due to their illness, we would had an accuracy of 10% for the sample of 96 subjects under study.

The sources of information used during the study were the patients’ clinical records and an ad hoc data collection log created for this study. Validated tools to determine the mental states of the patients (the MMSE [17], where lower scores indicate a worse mental state), assess anxiety and depression (the HADS [18], where higher scores indicate higher levels of anxiety and depression), and assess quality of life associated with urinary problems (the King’s Health Questionnaire [19], in which higher scores indicate worse quality of life) were also included. A separate questionnaire created by the researchers was included, which assessed the patients’ difficulties in performing IBC, relating specifically to frequency and intensity. The Cronbach’s alpha of this questionnaire was 0.895.

The outcome variables were maintenance of the number of IBCs prescribed by the professional (adherence), difficulties observed during the use of IBC, changes in quality of life (the King’s Health Questionnaire), and changes in mood (the HADS). The independent variables were made up of the patient’s sociodemographic, anthropometric, and clinical variables, previous mental state determinants, previous anxiety, previous depression, and previous quality of life.

Once the urologist had assessed the patient, by means of the relevant functional tests, the voiding diary, and as many examinations as necessary, the decision was made to prescribe bladder-emptying treatment by catheterization. The patient was then referred to the functional urology nurse, who indicated IBC and the number of daily catheterizations to be performed based on all previous tests.

Therefore, in order to determine if the patient was adherent to IBC, they were asked if they complied with the number of catheterizations prescribed when the treatment was indicated. Using a consecutive non-probability sampling method, the nursing researchers at the participating facilities invited patients who had just received IBC prescription to participate in the study, informing them of all the study’s details.

After accepting and signing the informed consent form, they were asked for additional information, which was usually not recorded in their clinical records.

The nurses carried out their work of informing the patients and training them to comply with IBC treatment, following the usual guidelines employed in each department. At that time, they filled in the data collection log, along with the HADS, MMSE, and the King’s Health Questionnaire.

The nurses contacted the patients by telephone every month to find out if they were following their IBC treatment and their reason for stopping the treatment, if that was the case. The monitoring log, the quality of life questionnaire (King’s Health Questionnaire), the anxiety and depression status questionnaire (HADS), and the questionnaire about difficulties with IBC were filled in at that time.

### 2.2. Statistical Analysis Used

First, descriptive statistical analyses were conducted on all of the study variables, using absolute and relative frequencies for qualitative variables and means with standard deviations for quantitative variables that followed a normal distribution; failing that, medians with interquartile ranges were used.

The changes in quality of life and the changes in anxiety/depression levels between baseline and each monthly report were evaluated using the Student–Fisher *t*-test for paired data. The SPSS 24.0 statistical package was used for all of the analyses.

### 2.3. Legal and Ethical Considerations

This observational study was carried out on anonymous data and has been designed in accordance with the Declaration of Helsinki, enacted by the World Medical Association (WMA). It was submitted to the Clinical Research Ethics Committee (CEIC) of the recruiting hospitals.

The patients were invited to participate in the study. They were given an information sheet, the study was explained to them orally, and they signed the informed consent form. The patients’ personal data were encoded using a numerical code to ensure the confidentiality of this information, in case they wanted to be part of the study.

In legal terms, the Organic Law 5/2018 on Personal Data Protection guarantees the anonymity of the participants and the database, and no personal data can be used to identify them.

## 3. Results

A total of 99 subjects participated in the study. Among the most relevant sociodemographic characteristics was the fact that the patients had a mean age of 35.2 years, which varied greatly from 18 years to 83 years. In total, 53.5% of the sample consisted of men, 41.4% of whom had completed primary education and 45.5% were retired. The vast majority lived at home with family and/or a carer (90.8%). The rest of the information can be found in Table 1.

With regard to clinical characteristics, it was highlighted that 32.3% of the subjects had neurological damage, due to which they were prescribed IBC, and 27.3% had impaired contractile function. Among the previous diseases suffered, cardiovascular diseases were the most prevalent, at 22.2%, followed by musculoskeletal diseases at 21.1%. Among the comorbidities collected, obesity stood out, at 17.2%, followed by previous episodes of depression, at 12.1%. In 89.9% of the patients, the catheter was prescribed by a urologist, and in 13.1% it was prescribed by a nurse. The rest of the characteristics can be found in Table 2.

With regard to self-care, the functionality of the subjects’ hands was assessed, noting that only 3% had limited motor skills in both hands, another 3% had only the dominant hand affected, and 11.1% had an impact on their sensitivity. Mobility was also assessed, finding that only 56.6% had normal mobility. On the other hand, 20.2% of the patients recognized that they found it difficult to see the urinary meatus. Most patients were able to repeat the information provided and understand it, at 97.0% and 92.9%, respectively. Interestingly, in this first assessment, 86.9% of patients believed that they would need help to carry out IBC. The rest of the information can be found in Table 3.

They were then asked about their concerns about using IBC. In this regard, a percentage value was obtained according to the degree of concern using a Likert scale ranging from “No concern” to “Very concerned”. Focusing on the “Very concerned” category, the factor that causes most concern among the patients was the risk of infection, with 6.1%. This factor also attained the highest mean and median scores, considering all response options together. As for the “No concern” category, the factor with the highest response percentage was loss of masculinity and femininity, at 69.7%, also attaining the lowest mean and median scores. The rest of the information can be found in Table 4.

In terms of mental state, 90.9% had normal values and only one person showed scores consistent with dementia. In terms of quality of life, the most affected aspects were impact (question 2 of the questionnaire) and personal relationships, while a prevalence of 14.1% of anxiety cases and 8.1% of depression cases was observed. The rest of this kind of information can be found in Table 5.

After a month of monitoring, the researchers contacted the patients to assess their status, determine whether they were continuing with the IBC, and to ask how their experience had been, and how it had affected their quality of life and their levels of anxiety and depression. All information can be found in Table 6.

First, they were asked about the type of catheter they had been using, observing that one in three patients used a ready-to-use hydrophilic catheter. During this period, one subject withdrew from the study due to a change of facility and three subjects left because their treatment ended. Some of the most interesting results include that 67.7% of patients performed IBC autonomously, despite the fact that at the start of the study most believed that they would need help, and 86.7% adhered to the number of prescribed catheterizations.

Other aspects analyzed were the difficulties the patients had experienced in performing bladder catheterization, in terms of both intensity and frequency. Although a Likert question was originally posed with four response options (0–3 points), the mean score of each proposed difficulty was calculated to derive an overall view of the most affected aspects. Specifically, the aspects with the highest mean scores were “The public bathrooms did not meet hygienic requirements to perform the catheterization”, followed by “I could not find a private place to perform the catheterization in public spaces”, in terms of both frequency and intensity. All information on IBC difficulties can be found in Table 7.

Finally, the changes in the quality of life and the anxiety–depression scale were evaluated after the first month. For this analysis, the baseline (recruitment visit) was compared with the first visit (*n* = 79). As shown in Table 8, an improvement in quality of life (lower King’s scores) was observed in all aspects, with the exception of personal relationships, as well as in anxiety and depression levels, at visit T1 compared to T0.

## 4. Discussion

Among the most relevant results of this study were that the profile of people requiring IBC varied greatly in sociodemographic and clinical characteristics, with neurological damage as the reason for prescription in one in three patients. This variability in the prescriptions and characteristics of patients requiring IBC can also be seen in other works [20,21].

On the other hand, although the majority of participants in our study had a good cognitive status and understood the instructions for conducting IBC well, more than half of the patients had mobility problems, and one in five found it difficult to properly see the urinary meatus. In this sense, we believe it is important for patients to participate in choosing the most appropriate IBC material according to their clinical and social circumstances, based on the training and information provided by the expert nurses in this field. In fact, some research considers the nurse’s recommendation as the most influential factor in the choice of IBC material, followed by the convenience of its use. Therefore, it is essential that the nurses in charge of these departments be highly qualified [22]. The patient’s active participation in choosing the catheter, their training and close monitoring by nurses specialized in performing this technique, and their integration of the treatment into their everyday life are the determining factors of adherence to the treatment [22].

Furthermore, patients in our study reported using seven types of catheters, with different materials and technical characteristics, which demonstrates the great variability in individual needs. The choice of one type of catheter or another is intended to promote adherence to IBC and avoid complications, depending on the preferences and clinical characteristics of each patient. In this regard, limiting the choice of catheter could pose a risk to adherence to IBC in the early stages, and could lead to the appearance of possible complications, such as hematuria or urethral bleeding. It is especially important to properly train patients and personalize their treatments at the beginning, because fear of the first IBC is common among patients [14]. In this regard, another result observed in our study regarding patients’ concerns is noteworthy. Specifically, the fear of infection was their greatest concern, while loss of masculinity and femininity was the least concerning issue for them. It is possible that this result could change with long-term follow-up. Once patients master IBC and overcome their fear of infection, issues relating to sexuality become more relevant.

Among the greatest difficulties and/or barriers expressed by the patients, they highlighted that “The public bathrooms did not meet hygienic requirements to perform the catheterization”, followed by “I could not find a private place to perform the catheterization in public spaces”, in terms of both frequency and intensity. Similar barriers have been observed by other authors, such as Cobussen et al. [13]. In their study, they found that patients were particularly concerned about preparation before the technique and the need to plan the timing of catheterization. These factors demonstrate once again that patients are able to use catheterization materials that allow them to perform the IBC technique under conditions that are as aseptic as possible, without any previous steps and in a simple way. However, fear of these situations could seriously condition those affected by limiting their movement, travel and leisure activities outside their homes, and, in short, their ability to lead a normal life.

Despite this variability in both the profile of patients who use IBC and the difficulties observed, there is a high percentage of adherence to IBC (86.7%), which is higher than that observed by other authors, such as Hentzen et al. [23], who observed 66.9%, and Montavaselli et al. [24], who observed 29% after the month of monitoring. We also observed a significant change between the initial perception of needing support to conduct catheterization and the degree of independence in self-catheterization achieved after the first month. From the patients’ initial perception of their ability to perform self-catheterization, which they rated at 13%, to one month later and after the training carried out with expert nurses, 68% were able to take control of their treatment autonomously and perform self-catheterizations. In this regard, we believe that the success of this outcome is the result of the significant involvement of nurses specialized in patient training.

Regarding the influence of IBC on quality of life and mood, we observed a significant improvement in the short-term of quality of life, coinciding with other authors [12], although these same authors also recognized that it is important that adequate support be provided in the early stages by trained nurses, especially with elderly people. An improvement in anxiety–depression levels was also observed, which is a very interesting result, since higher levels of anxiety–depression have been associated with a lower adherence to IBC [24]. This result, which shows a decrease in mood issues, could be partly attributed to the training the nurses carried out with patients for the use of IBC, as other authors have also observed [15].

Among the main limitations of our study, we note that it was carried out with short-term monitoring. There was also a loss of 20 subjects as a result of the COVID-19 pandemic. However, despite these limitations, our study recruited more subjects than most published papers, the majority of which are qualitative in nature [12,13,14,15,16]. Another limitation observed is that the net effect of training on adherence cannot be determined, because although the training is based on protocols, it is individualized and adapted to the situation of each patient. Thus, it is not possible to separate the influence of training from the influence that the individual characteristics of each patient have on adherence.

Among its main strengths, our study is significant because it covers a topic with little international coverage in the literature, and it is the first to address this problem in Spain with a multicenter approach, whereby patients from 24 Spanish hospitals are represented. The selection of subjects has been rigorous and systematic. Therefore, we believe that the confounding bias may be minimal. Validated tools have also been used in the determination of the phenomena under study.

Although adherence rates of 90% were achieved initially, we must be cautious and conservative, and although the results are positive, the investigators consider it necessary to increase the number of subjects under study to be able to detect events or factors that influence adherence, but with less weight, thus improving statistical power.

On the other hand, it is well known that adherence can be at its highest during the first visits, and subsequently decrease, so we consider it necessary to continue with the study beyond the recruitment of subjects into long-term follow-ups. Additionally, based on the experience obtained in this pilot study, the researchers believe it appropriate to include in the assessment of new patients and throughout the follow-up some new indicator of adequate adherence, which operates both subjectively, manifested through the sensations of the patients, and objectively, through urodynamic tests.

Implications for practice:

Based on the observed results, we consider that individualized patient care is necessary, and that the adequate training of patients by specialized professionals and patient participation in decision-making are essential to favoring adherence to IBC treatment.

Given that the risk of infection is one of the issues of greatest concern to patients once they start IBC treatment, nurses should place special emphasis on this aspect, explaining to the patient the preventive measures to be taken and the fact that it is not necessary to abandon treatment because of infection, thus promoting adherence to IBC.

Given that the most significant difficulties reported by patients in performing catheterization outside their home were the lack of hygiene and privacy in public bathrooms, it is essential that they are able to select the materials that will enable them to perform the technique under the best conditions, and thus to guarantee adherence to IBC.

## 5. Conclusions

In this first stage of piloting, there is good adherence to IBC, with a significant percentage of personal autonomy when performing self-catheterization. In our study population, a significant improvement in the patients’ quality of life and mood also occurred after one month of monitoring. These results could largely be attributed to adequate patient training, and the adequate personalization of the IBC treatment and materials, by the nurses specialized in functional urology. With the extension of this study to 1 year of monitoring, we hope that these positive results at the start of the treatment will last over time, ensured by the close monitoring of the nurses specialized in functional urology.

## Figures and Tables

**Table 1:** Sociodemographic characteristics of the patients who participated in the study.

Variable	Starting Cohort *n* (%) *n* = 99
Age in years (Mean ± SD)	35.2 (20.5)
Sex	
Male	53 (53.5)
Female	46 (46.5)
Level of education	
No education	4 (4.0)
Primary education	41 (41.4)
Secondary education	26 (26.3)
University	28 (28.3)
Employment status	
Retired	45 (45.5)
On leave	23 (23.2)
Leave of absence	1 (1.0)
Unemployed	8 (8.1)
Employed	22 (22.2)
Marital status	
Married	64 (64.6)
Divorced	3 (3.0)
Separated	2 (2.0)
Single	25 (25.3)
Window(er)	5 (5.1)
Residence	
Lives at home alone	8 (8.1)
Lives at home with family and/or carer support	90 (90.8)
Lives at a nursing home	1 (1.0)

**Table 2:** Clinical characteristics of patients under study.

Variable	*n* (%) *n* = 99
Situation leading to IBC prescription:	
Postoperative bladder dysfunction	16 (16.2)
Impaired contractile function (no neurological disorder)	27 (27.3)
Neurological damage	32 (32.3)
Neobladder	7 (7.1)
Infravesical obstruction (benign prostatic hyperplasia, prolapse)	2 (2.0)
Neurodegenerative disease (sclerosis)	10 (10.1)
Bladder sphincter dyssynergia	2 (2.0)
Pre-existing conditions	
Cardiovascular disease	22 (22.2)
Neurological disease	17 (17.2)
Endocrine disease	18 (18.2)
Respiratory disease	6 (6.1)
Gastrointestinal disease	7 (7.1)
Genitourinary diseases	20 (20.2)
Musculoskeletal disease	21 (21.2)
Psychiatric illness	7 (7.1)
Comorbidities	
Obesity	17 (17.2)
Prolapse	1 (1.1)
Benign prostatic hyperplasia	10 (10.1)
Bladder spasm	1 (1.1)
History of depression	12 (12.1)
History of anxiety	11 (11.1)
Person who prescribed bladder catheterization (may be more than one)	
Nurse	13 (13.1)
Urologist	89 (89.9)
Gynecologist	0 (0.0)
Rehabilitation therapist	7 (7.1)
Neurologist	0 (0.0)

**Table 3:** Characteristics related to self-care capability.

Variable	*n* (%) *n* = 99
Hand function as reported by the patient	
Normal	82 (82.8)
Limited sensitivity, but with normal motor skills	11 (11.1)
Limited motor skills in the dominant hand	3 (3.0)
Limited motor skills in the non-dominant hand	0 (0.0)
Limited motor skills in both hands	3 (3.0)
Mobility as reported by the patient	
Normal	56 (56.6)
Difficulty in walking, but does not require help	15 (15.2)
Can walk with help	7 (7.1)
Walks with a wheelchair, but possible transfer on foot	8 (8.1)
Permanently in a wheelchair	13 (13.1)
Difficulty in seeing urinary meatus	
No	79 (79.8)
Yes	20 (20.2)
The patient can repeat the information on IBC provided by the nurse	
No	1 (1.0)
Yes	96 (97.0)
Unsure	2 (2.0)
The patient can follow the instructions given by the nurse	
No	2 (2.0)
Yes	92 (92.9)
Unsure	5 (5.1)
Who do you think is going to perform the IBC	
Me (self-catheterization)	13 (13.1)
With the help of another person (assisted)	86 (86.9)

**Table 4:** Patients’ degree of concern over different problems that could be attributed to catheterization at the first visit.

*n* = 99	No concern	A Little Concerned	Somewhat Concerned	Quite Concerned	Very Concerned	Md (IQR)	*M* (SD)
About inserting the catheter into their body	47 (47.5)	16 (16.2)	18 (18.2)	15 (15.2)	3 (3.0)	2.0 (2)	2.1 (1.24)
About getting an infection	26 (26.3)	14 (14.1)	32 (32.3)	21 (21.2)	6 (6.1)	3.0 (3)	2.7 (1.24)
About pain during catheterization	44 (44.4)	16 (16.2)	23 (23.2)	11 (11.1)	5 (5.1)	2.0 (2)	2.2 (1.25)
About suffering an injury to the urethra	34 (34.3)	18 (18.2)	29 (29.3)	13 (13.1)	5 (5.1)	2.0 (2)	2.4 (1.22)
About loss of dignity	61 (61.6)	25 (25.3)	9 (9.1)	2 (2.0)	2 (2.0)	1.0 (1)	1.6 (0.89)
About loss of masculinity or femininity	69 (69.7)	22 (22.2)	6 (6.1)	2 (2.0)	0 (0.0)	1.0 (1)	1.4 (0.69)
About social rejection	67 (67.7)	21 (21.2)	6 (6.1)	3 (3.0)	2 (2.0)	1.0 (1)	1.5 (0.89)
About losing control of themself	55 (55.6)	24 (24.2)	15 (15.2)	5 (5.1)	0 (0.0)	1.0 (1)	1.7 (0.91)

Md: Median; IQR: Interquartile range; M: Mean; SD: Standard deviation.

**Table 5:** Cognitive characteristics, quality of life, and state of mind of the patients under study at first visit.

Variable	Mean (SD)	*n* (%) *n* = 99
Mental state. Mini-Mental State Examination (MMSE)	31.7 (4.77)	
Normal (27 or more)		91 (91.9)
Questionable (24–27 points)		2 (2.0)
Deterioration (12–24 points)		5 (5.1)
Dementia (<12 points)		1 (1.0)
Quality of life. King’s Health Questionnaire Dimensions		
General health perception	39.7 (21.16)	
Incontinence impact	58.9 (32.93)	
Role limitations	37.2 (33.06)	
Physical limitations	36.5 (36.63)	
Social limitations	29.7 (31.80)	
Personal relationships	55.7 (33.41)	
Emotions	28.2 (26.52)	
Sleep/Energy	28.1 (32.61)	
Severity measures	35.3 (26.84)	
State of mind. Hospital Anxiety and Depression Scale (HADS)		
Anxiety score	6.1 (4.58)	
Level of anxiety		
Normal		71 (71.4)
Borderline case		14 (14.1)
Case		14 (14.1)
Depression score	4.6 (4.10)	
Level of depression		
Normal		82 (82.8)
Borderline case		9 (9.1)
Case		8 (8.1)

**Table 6:** Characteristics of the catheter used and its method of use. First visit.

Variable	*n* (%) *n* = 79
Type of catheter	
Hydrophilic catheter requiring internal activation or other pre-catheterization step (break bag of built-in solution, unscrew connector, remove fluid from container, etc.)	10 (10.1)
Hydrophilic catheter requiring internal activation or other pre-catheterization step (breaking bag of built-in solution, unscrew connector, remove fluid from container, etc.) with integrated diuresis bag	3 (3.0)
Catheter pre-lubricated with gel and with an integrated diuresis bag	4 (4.0)
Pre-lubricated hydrophilic ready-to-use catheter (with internal solution without activation required)	21 (21.2)
Pre-lubricated hydrophilic ready-to-use catheter (with internal solution without activation required) with integrated diuresis bag	2 (2.0)
Pre-lubricated hydrophilic ready-to-use catheter (with Vaporphilic Technology)	34 (34.3)
Pre-lubricated hydrophilic ready-to-use catheter with integrated diuresis bag (with Vaporphilic Technology)	5 (5.1)
End of treatment	3 (3.0)
Change of facility	1 (1.0)
Who performs the catheterization	*n* = 79
The patient	67 (67.7)
Their partner	9 (10.7)
Another family member	4 (4.0)
External carer	1 (1.0)
Patient continues IBC	*n* = 79
No	4 (5.1)
Yes	75 (94.9)
Patient conducts number of catheterizations prescribed (adherent)	*n* = 75
No	10 (13.3)
Yes	65 (86.7)

**Table 7:** Difficulty performing bladder catheterization.

	Frequency *n* = 79					Intensity *n* = 79				
	Never *n* (%)	Sometimes *n* (%)	Often *n* (%)	Always *n* (%)	Mean (SD)	No *n* (%)	A little *n* (%)	Moderate *n* (%)	Considerable *n* (%)	Mean (SD)
I’ve experienced pain.	47 (59.5)	28 (35.4)	4 (5.1)	0 (0.0)	0.46 (0.60)	46 (58.2)	30 (38.0)	3 (3.8)	0 (0.0)	0.46 (0.57)
I’ve experienced bleeding.	61 (77.2)	18 (22.8)	0 (0.0)	0 (0.0)	0.23 (0.42)	61 (77.2)	16 (20.3)	2 (2.5)	0 (0.0)	0.25 (0.49)
I can identify the meatus.	54 (68.4)	19 (24.1)	5 (6.3)	1 (1.3)	0.41 (0.67)	54 (68.4)	20 (25.3)	4 (5.1)	1 (1.3)	0.39 (0.65)
I can open the catheter container.	76 (96.2)	3 (3.8)	0 (0.0)	0 (0.0)	0.04 (0.19)	76 (96.2)	3 (3.8)	0 (0.0)	0 (0.0)	0.04 (0.19)
Activation/preparation of the catheter.	76 (96.2)	3 (3.8)	0 (0.0)	0 (0.0)	0.04 (0.19)	76 (96.2)	3 (3.8)	0 (0.0)	0 (0.0)	0.04 (0.19)
Conduct self-catheterization with “no touch” technique (prevent risk of bacterial contamination).	59 (74.7)	19 (24.1)	1 (1.3)	0 (0.0)	0.27 (0.47)	59 (74.7)	18 (22.8)	2 (2.5)	0 (0.0)	0.28 (0.51)
Conduct self-catheterization (hardness or flexibility).	68 (86.1)	9 (11.4)	2 (.5)	0 (0.0)	0.16 (0.44)	69 (87.3)	8 (10.1)	2 (2.5)	0 (0.0)	0.15 (0.43)
During catheterization (insertion, progress, and removal).	45 (57.0)	30 (38.0)	3 (3.8)	1 (1.3)	0.49 (0.64)	47 (59.5)	27 (34.2)	4 (5.1)	1 (1.3)	0.48 (0.66)
Conduct self-catheterization at social gatherings due to fear of spilling the container liquid onto myself.	62 (78.5)	11 (13.9)	6 (7.6)	0 (0.0)	0.30 (0.60)	62 (78.5)	11 (13.9)	6 (7.6)	0 (0.0)	0.29 (0.60)
The container’s lack of discreetness causes me to avoid catheterization when I am with other people.	66 (83.5)	11 (13.9)	1 (1.3)	0 (0.0)	0.20 (0.52)	66 (83.5)	10 (12.7)	1 (1.3)	2 (2.5)	0.23 (0.60)
The public bathrooms did not meet hygienic requirements to perform the catheterization.	38 (48.1)	16 (20.3)	17 (21.5)	8 (10.1)	0.94 (1.01)	40 (50.6)	14 (17.7)	6 (20.3)	9 (11.4)	0.92 (1.08)
Problems with accessing public bathrooms.	54 (8.4)	14 (17.7)	11 (13.9)	0 (0.0)	0.46 (0.73)	54 (68.4)	14 (17.7)	11 (13.9)	0 (0.0)	0.46 (0.73)
I could not find a private place.	43 (54.4)	18 (22.8)	12 (15.2)	6 (7.6)	0.76 (0.98)	44 (55.7)	17 (21.5)	12 (15.2)	6 (7.6)	0.75 (0.98)
I found it difficult to plan.	57 (72.2)	11 (11.9)	7 (8.9)	4 (5.1)	0.47 (0.86)	58 (58.6)	10 (12.7)	7 (8.9)	4 (5.1)	0.46 (0.86)
Lack of help.	76 (96.2)	3 (3.8)	0 (0.0)	0 (0.0)	0.04 (0.19)	76 (97.5)	2 (2.5)	0 (0.0)	0 (0.0)	0.03 (0.16)
Lack of time.	70 (88.6)	7 (8.9)	1 (1.3)	1 (1.3)	0.15 (0.48)	70 (88.6)	7 (8.9)	1 (1.3)	1 (1.3)	0.15 (0.8)

**Table 8:** Evolution of quality of life and mood during follow-up between T0 and T1.

Variable	Comparison between Recruitment (T0) and First Visit (T1) *n* = 79				
King’s Health Questionnaire Dimensions	Media (SD)	Mean (SD)	Difference in Means	95% CI	*p*-Value
General health perception	43.0 (19.2)	36.7 (20.0)	6.33	2.08; 10.58	0.004
Incontinence impact	58.2 (31.8)	43.5 (33.1)	14.77	7.52; 22.02	<0.001
Role limitations	36.7 (32.9)	28.1 (31.4)	8.65	2.11; 15.19	0.010
Physical limitations	35.4 (35.5)	26.2 (31.5)	9.29	2.40; 16.17	0.009
Social limitations	27.5 (30.2)	20.5 (28.6)	7.03	1.95; 12.12	0.007
Personal relationships	63.1 (32.8)	50.0 (30.7)	13.00	−8.97; 35.17	0.222
Emotions	27.3 (25.8)	15.6 (22.7)	11.67	5.89; 17.46	<0.001
Sleep/Energy	30.0 (31.5)	15.4 (30.9)	14.56	7.48; 21.64	<0.001
Severity measures	37.2 (27.3)	29.5 (25.7)	7.68	1.81; 13.55	0.011
HADS					
Anxiety	6.1 (4.1)	4.5 (3.69)	1.65	0.91; 2.37	<0.001
Depression	4.3 (3.86)	3.7 (3.90)	0.62	0.01; 1.23	0.046

## Data Availability

The data sets generated and/or analyzed during the current study are available from the corresponding author on reasonable request.
